# Fat Browning Effects of Catalpol and Rhoifolin from *Rehmannia glutinosa* (Gaertn.) and *Lonicera japonica* (Thunb.) in 3T3-L1 Adipocytes via the β3-AR Signaling Pathway

**DOI:** 10.3390/ph19050787

**Published:** 2026-05-18

**Authors:** Seung Min Choi, Sung Ho Lim, Ho Seon Lee, Gayoung Choi, Myeong Ji Kim, Hyunwoo Kim, Chang-Ik Choi

**Affiliations:** BK21 FOUR Team and Integrated Research Institute for Drug Development, College of Pharmacy, Dongguk University—Seoul, Goyang 10326, Republic of Korea; tlsehdhs@dgu.ac.kr (S.M.C.); sho617@dgu.ac.kr (S.H.L.); ghtjsrhtn@dongguk.edu (H.S.L.); gayoung@dgu.ac.kr (G.C.); mjk980302@dgu.ac.kr (M.J.K.)

**Keywords:** white adipose tissue browning, thermogenesis, plant-derived compounds

## Abstract

**Background/Objectives**: Promoting white adipose tissue (WAT) browning into thermogenic beige adipocytes is a promising anti-obesity strategy. *Yanggyeoksanhwa-tang* (YST) has been used traditionally to alleviate obesity-related conditions. Catalpol and rhoifolin are major bioactive components of *Rehmannia glutinosa* (Gaertn.) and *Lonicera japonica* (Thunb.) with known metabolic or anti-inflammatory effects. However, their direct roles in adipocyte browning and the mechanisms via β3-adrenergic receptor (β3-AR) signaling are not well defined, and this study addresses this gap. **Methods**: To evaluate browning potential, 3T3-L1 adipocytes were treated with catalpol and rhoifolin during differentiation. The expression of browning markers and lipid metabolism or catabolism transcription factors was analyzed using Western blotting and quantitative real-time polymerase chain reaction. The involvement of the β3-AR and adenosine monophosphate–activated protein kinase (AMPK) signaling pathways was further validated using specific agonists and antagonists. **Results**: Both compound treatments significantly upregulated beige-specific (*Cd137*, *Cited*, *Tbx1*, *Cidea*, *Fgf21*, *Tmem26*) and mitochondrial biogenesis markers (*Cox4*, *Nrf1*, *Tfam*), accompanied by a marked increase in thermogenic markers (UCP1, PGC-1α, *Prdm16*). Concurrently, lipolysis-related genes such as *Atgl*, *Hsl*, and *Plin1* were elevated, while lipogenesis targets (*Fasn*, *Lpl*, *Srebf1*, *Acaca*) were downregulated through activation of the β3-AR signaling pathway. **Conclusions**: These findings suggest that catalpol and rhoifolin, key phytochemicals of YST, promote WAT browning and lipolysis. Our findings indicate that these compounds induce browning and modulate metabolism via the β3-AR pathway. These results serve as a cornerstone for natural anti-obesity therapy, pending further validation in vivo and clinical studies.

## 1. Introduction

Obesity has become a global health problem due to the rapid adoption of Western diets and modernization [1]. Beyond being a disease, obesity is closely associated with metabolic disorders, including cardiovascular disease and nonalcoholic fatty liver disease [2,3]. To address this growing health burden, several therapeutic strategies have been proposed, including appetite suppression, inhibition of nutrient absorption, and increased energy expenditure [4,5]. However, multiple current pharmacological treatments, including appetite suppressants and nutrient absorption inhibitors, are often associated with significant side effects, such as gastrointestinal distress and cardiovascular risks [6]. Consequently, safer natural therapeutic agents that increase metabolic rate and energy expenditure are urgently needed.

Among these, the browning of white adipose tissue (WAT) has gained considerable attention as a promising strategy to increase energy expenditure [7]. While WAT primarily stores excess energy, research shows the therapeutic potential of beige adipocytes. These cells arise within WAT depots and exhibit thermogenic capacities similar to those of brown adipose tissue (BAT), representing a novel target for anti-obesity interventions through a process known as browning [8]. WAT is characterized based on few mitochondria and large unilocular lipid droplets. In contrast, BAT dissipates energy as heat generation (thermogenesis) due to its abundant mitochondria [9]. Uncoupling protein 1 (UCP1) in mitochondria is a key driver of thermogenesis. UCP1 uncouples oxidative phosphorylation by allowing protons to cross the mitochondrial inner membrane, resulting in heat production and increased energy expenditure [10]. Recent studies report that various natural compounds promote WAT browning by increasing mitochondrial UCP1 expression, highlighting the potential of herbal remedies as anti-obesity strategies [11,12,13,14,15]. Resveratrol (25 μM) increased UCP1 mRNA by approximately 250-fold in 3T3-L1 adipocytes (*p* < 0.05), capsaicin (1 μM) elevated UCP1 mRNA by >90-fold in 3T3-L1 adipocytes (*p* < 0.05) [11], and curcumin enhanced UCP1 in high-fat diet-fed C57BL/6J mice by 4-fold at the mRNA level and 5-fold at the protein level in BAT (*p* < 0.05) [13]. Thus, fat browning, which converts WAT into a BAT-like state, may represent a therapeutic strategy for obesity.

**Figure 1:**
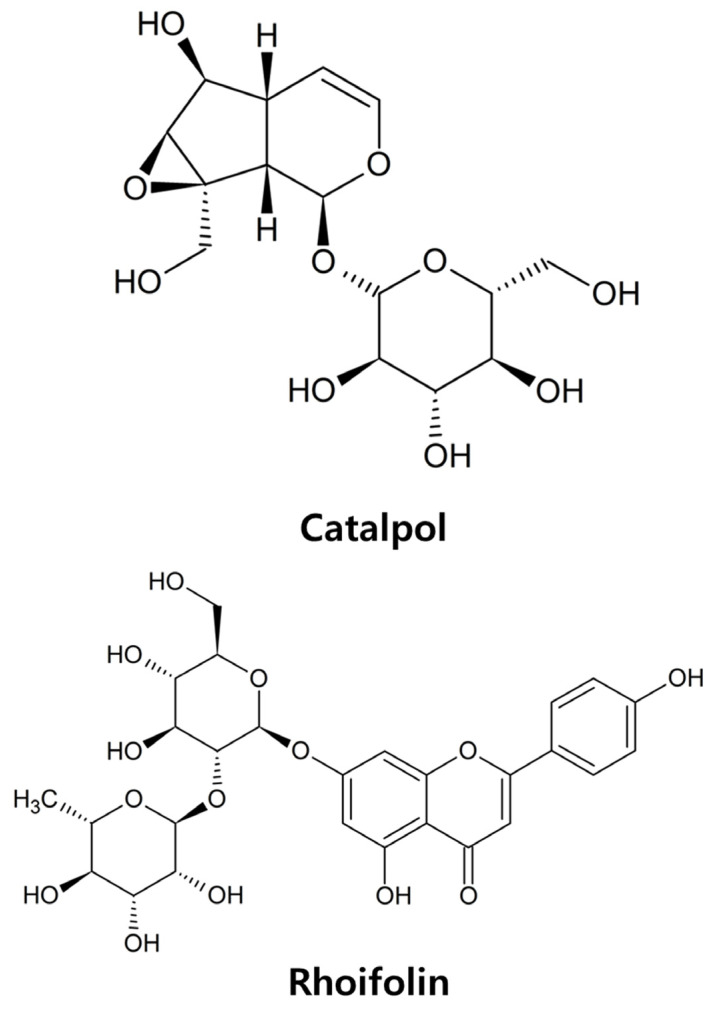
Chemical structures of catalpol and rhoifolin.

In traditional medicine, *Yanggyeoksanhwa-tang* (YST) has been used to alleviate inflammation and obesity [16], corresponding in modern clinical terms as metabolic overactivation and chronic inflammation associated with obesity [17]. Its primary components, *Rehmannia glutinosa* (Gaertn.) (*R. glutinosa*) and *Lonicera japonica* (Thunb.) (*L. japonica*), belong to the Scrophulariaceae and Caprifoliaceae families, respectively [18,19]. The dried root of *R. glutinosa* contains active constituents such as terpenoids, flavonoids, and lignans and exhibits various medicinal properties [20]. These compounds exhibit pharmacological activities, including antidiabetic, anti-inflammatory, anticancer, and immunomodulatory effects [18,21]. *L. japonica* has been used in traditional medicine to treat fevers, wounds, abscesses, boils, and some infectious diseases [22]. Modern pharmacological research shows that *L. japonica* and its active compounds exhibit diverse pharmacological activities, including antibacterial, antidepressant, antiviral, anti-inflammatory, and antipyretic effects [20,23,24,25,26,27]. Rhoifolin (4′,5-Dihydroxy-7-[α-L-rhamnopyranosyl-(1→2)-β-D-glucopyranosyloxy]flavone), a common flavonoid isolated from *L. japonica* [28,29], is used to manage chronic diseases such as diabetes and obesity [30]. This compound demonstrates antioxidant, anti-inflammatory, anticancer, and hepatoprotective properties. While its efficacy in obesity-related diseases is recognized, studies exploring its role in fat browning remain limited [31,32,33]. Furthermore, while catalpol ((1a*S*,1b*S*,2*S*,5a*R*,6*S*,6a*S*)-6-Hydroxy-1a-(hydroxymethyl)-1a,1b,2,5a,6,6a-hexahydrooxireno [2′,3′:4,5]cyclopenta [1,2-*c*]pyran-2-yl β-D-glucopyranoside) from *R. glutinosa* improves glucose metabolism, its specific effect on thermogenic gene expression, alone or combined with rhoifolin, remains unclear. Therefore, this study aims to investigate whether these herbs, and the active components, catalpol and rhoifolin (Figure 1), induce browning-like remodeling and metabolic activation in 3T3-L1 adipocytes, and to evaluate the involvement of the β3 adrenergic receptor (β3-AR) and adenosine monophosphate–activated protein kinase (AMPK) pathways in these processes.

## 2. Results

### 2.1. Effects of Single Herbs and Their Active Compounds in YST on Cell Viability and Lipid Accumulation in 3T3-L1 Adipocytes

**Figure 2:**
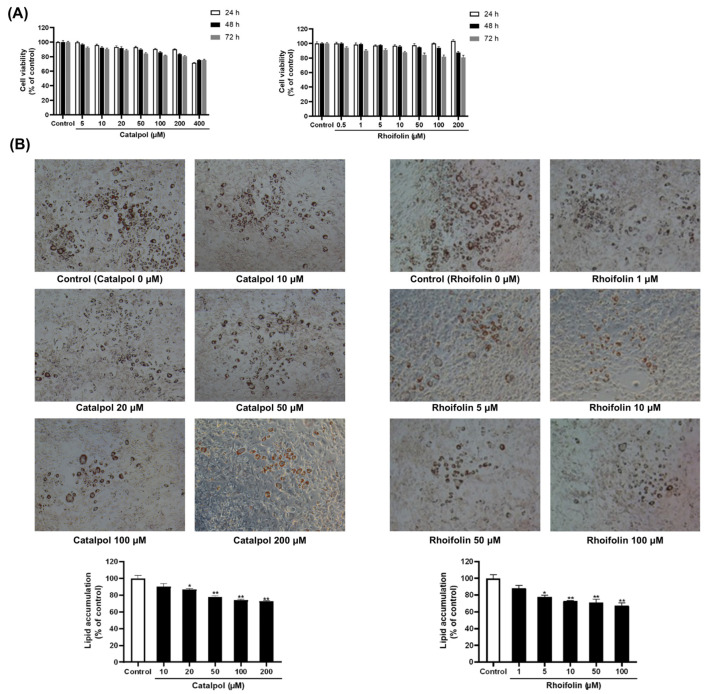
Effects of catalpol and rhoifolin on cell viability and lipid accumulation in 3T3-L1 adipocytes. (**A**) Cell viability was examined after 24 h, 48 h, and 72 h of treatment using the MTT assay. Cells were seeded in 96-well plates and incubated for 24 h prior to treatment. Results are expressed as a percentage of the control (0.5% Dimethyl sulfoxide, DMSO) (*n* = 6). (**B**) Lipid accumulation was evaluated after 7 days of differentiation. Cells were seeded in 24-well plates, with lipid droplets stained with Oil Red O, extracted using isopropanol, and quantified at 520 nm using a microplate reader. Representative cell images were obtained at 100× magnification. Statistical analysis was performed using Student’s *t*-test for comparisons between the control and each treatment group. Data are expressed as a percentage of the control (0.5% DMSO) and reported as mean ± standard error of the mean (SEM) from triplicates. * *p* < 0.05, and ** *p* < 0.01 vs. control.

3-(4,5-dimethylthiazol-2-yl)-2,5-diphenyltetrazolium (MTT) assay was conducted in 3T3-L1 preadipocytes to evaluate the effect of individual herbs and the active constituents (catalpol and rhoifolin) derived from YST on cell viability. Figure S1A shows that 3T3-L1 cells were treated with graded concentrations (1, 5, 10, 25, 50, 75, and 100 μg/mL) of *R. glutinosa* (RGE) and *L. japonica* (LJE) extracts for 24, 48, and 72 h. Both RGE and LJE maintained approximately 90% cell viability following 72 h of incubation at the highest tested concentration (100 μg/mL). Additionally, the effects of their active compounds (catalpol and rhoifolin) on cell viability were evaluated. Rhoifolin maintained approximately 80% cell viability after 72 h of exposure at the highest test concentration (200 μM), while catalpol reduced cell viability to <80% at 400 μM (Figure 2A). Based on these safety profiles, non-cytotoxic concentrations were selected for subsequent experiments. The effects of the individual herbs in YST and their components on lipid accumulation were investigated in 3T3-L1 preadipocytes differentiated for 7 days. RGE and LJE (1–100 μg/mL) effectively reduced lipid droplets size compared to the control (Figure S1B). Furthermore, catalpol and rhoifolin effectively inhibited lipid accumulation across the 1–200 μM concentration range (Figure 2B).

### 2.2. Effects of Single Herbs and Their Active Compounds in YST on Fat Browning Through Thermogenesis in 3T3-L1 Adipocytes

**Figure 3:**
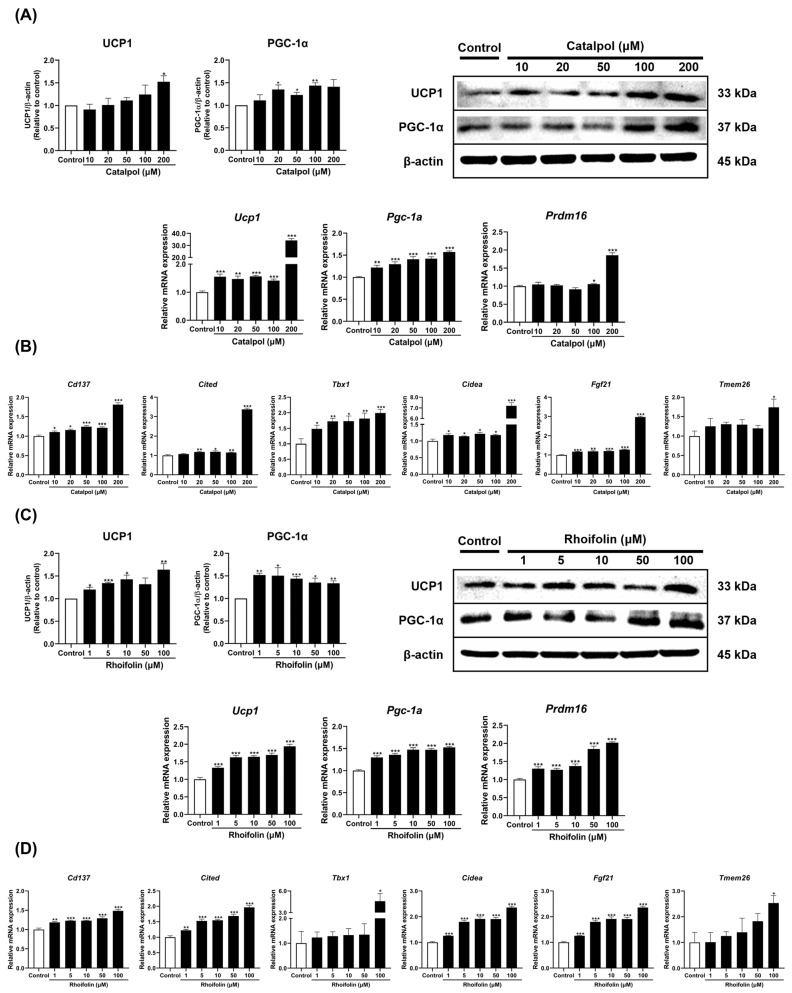
Effects of catalpol (**A**,**B**) and rhoifolin (**C**,**D**) on the expression of thermogenic and beige fat-specific markers in 3T3-L1 adipocytes. Target gene mRNA levels were normalized to Gapdh using the 2^−ΔΔCt^ method (*n* = 6). Gapdh was used as the housekeeping gene, while β-actin served as the loading control for protein expression (*n* = 3). Statistical analysis was performed using Student’s *t*-test for comparisons between the control and each treatment group. Results are presented as mean ± standard error of the mean (SEM). * *p* < 0.05, ** *p* < 0.01, and *** *p* < 0.001 vs. control.

The inhibitory effects of single herb extracts and their active constituents in modulating lipid accumulation through adipocyte browning were evaluated by analyzing key thermogenic markers. RGE and LJE extracts effectively upregulated the mRNA expression of *Ucp1*, *Pgc-1a*, and *Prdm16*, which are essential regulators of the thermogenic program. Furthermore, these extracts significantly enhanced the expression of beige-specific markers, including *Cd137*, *Cidea*, *Cited*, and *Fgf21*, confirming the induction of adipocyte browning (Figure S2). Catalpol and rhoifolin stimulated the transcription of thermogenic genes and significantly increased the protein levels of UCP1 and Peroxisome proliferator-activated receptor gamma co-activator 1α (PGC-1α) (Figure 3A,C). In 3T3-L1 adipocytes, treatment with these compounds markedly elevated the expression of beige-specific markers, such as *Cd137*, *Cidea*, *Cited*, *Fgf21*, *Tbx1*, and *Tmem26* at the highest tested concentrations (Figure 3B,D).

### 2.3. Effects of Single Herbs and Their Active Compounds in YST on Mitochondrial Biogenesis in 3T3-L1 Adipocytes

**Figure 4:**
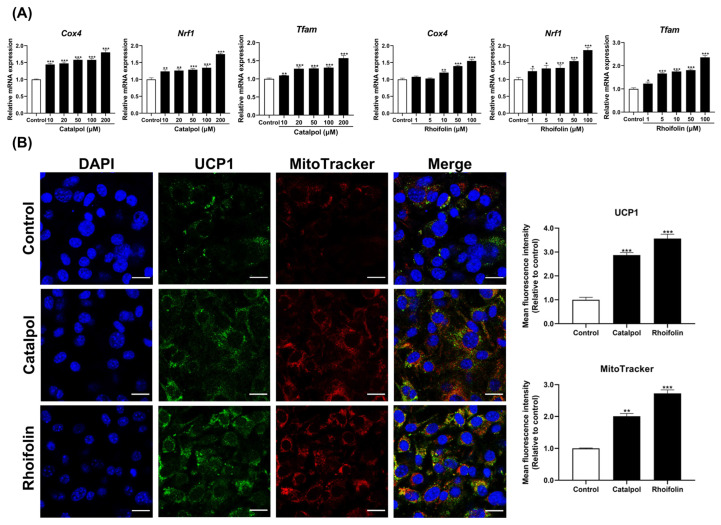
Effects of catalpol and rhoifolin on mitochondrial biogenesis in 3T3-L1 adipocytes. (**A**) mRNA expression of mitochondrial biogenesis markers was evaluated using qRT-PCR. *Gapdh* was used as the housekeeping gene, and target gene expression was normalized using the 2^−ΔΔCt^ method (*n* = 6). (**B**) Effects of their active compounds on intracellular mitochondrial biogenesis and UCP1 activation, as evaluated by using immunofluorescence staining and intensity analysis (*n* = 3). UCP1 protein was visualized using Fluorescein isothiocyanate (FITC) conjugated antibody (UCP1-FITC), 4′,6-Diamidino-2-phenylindole (DAPI, nuclei), and MitoTracker Red (mitochondria). Images were obtained at 60× magnification (scale bar = 10 μm). Statistical analysis was performed using Student’s *t*-test for comparisons between the control and each treatment group. Results are presented as mean ± standard error of the mean (SEM). * *p* < 0.05, ** *p* < 0.01, and *** *p* < 0.001 vs. control.

Given that an increase in mitochondrial content within WAT indicates a shift toward a BAT-like phenotype, mitochondrial biogenesis marker, such as *Cox4*, *Nrf1*, and *Tfam,* were analyzed. Specifically, mRNA analysis was conducted to investigate the effects of the individual herb extracts (RGE and LJE) and their active compounds (catalpol and rhoifolin). Both the extracts (Figure S3A) and their active compounds (Figure 4A) significantly upregulated mitochondrial marker genes, supporting the induction of browning in 3T3-L1 adipocytes. Immunofluorescence staining further demonstrated that high-dose treatments with these extracts (Figure S3B) and compounds (Figure 4B) significantly enhanced mitochondrial content and UCP1 protein levels compared to the control group.

### 2.4. Effects of Catalpol and Rhoifolin on Lipid Metabolism in 3T3-L1 Adipocytes

**Figure 5:**
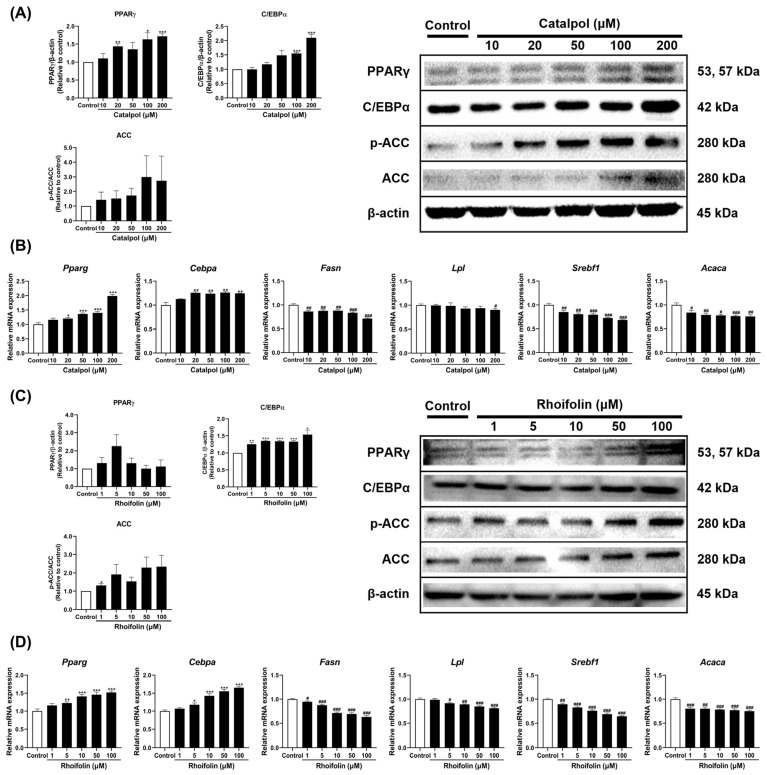
Effects of catalpol (**A**,**B**) and rhoifolin (**C**,**D**) on the expression of adipogenic and lipogenic markers in 3T3-L1 adipocytes. Target gene mRNA expression was analyzed using qRT-PCR and normalized to *Gapdh* using the 2^−ΔΔCt^ method (*n* = 6). *Gapdh* was used as the housekeeping gene, while β-actin served as the loading control (*n* = 3). Statistical analysis was performed using Student’s *t*-test for comparisons between the control and each treatment group. Data are expressed as mean ± standard error of the mean (SEM). * *p* < 0.05, ** *p* < 0.01, and *** *p* < 0.001 vs. control (increase); # *p* < 0.05, ## *p* < 0.01, and ### *p* < 0.001 vs. control (decrease).

Catalpol and rhoifolin were evaluated for their effects on the expression of key genes and proteins regulating adipogenesis, lipogenesis, and overall lipid metabolism. Catalpol treatment significantly increased both protein and mRNA levels of Peroxisome proliferator-activated receptor γ (PPARγ) and CCAAT/enhancer binding protein α (C/EBPα) (Figure 5A). Rhoifolin treatment specifically increased protein and mRNA levels of C/EBPα without affecting PPARγ (Figure 5C). Phosphorylation levels of acetyl-CoA carboxylase (ACC) exhibited an increasing trend; however, the changes induced by both compounds were not statistically significant (Figure 5A,C). Both compounds significantly decreased the expression of lipogenesis-related genes, such as *Fasn*, *Lpl*, *Srebf1*, and *Acaca* (Figure 5B,D). These findings indicate that catalpol and rhoifolin promote the formation of beige adipocytes while effectively suppressing lipogenesis.

### 2.5. Effects of Catalpol and Rhoifolin on Lipid Catabolism in 3T3-L1 Adipocytes

**Figure 6:**
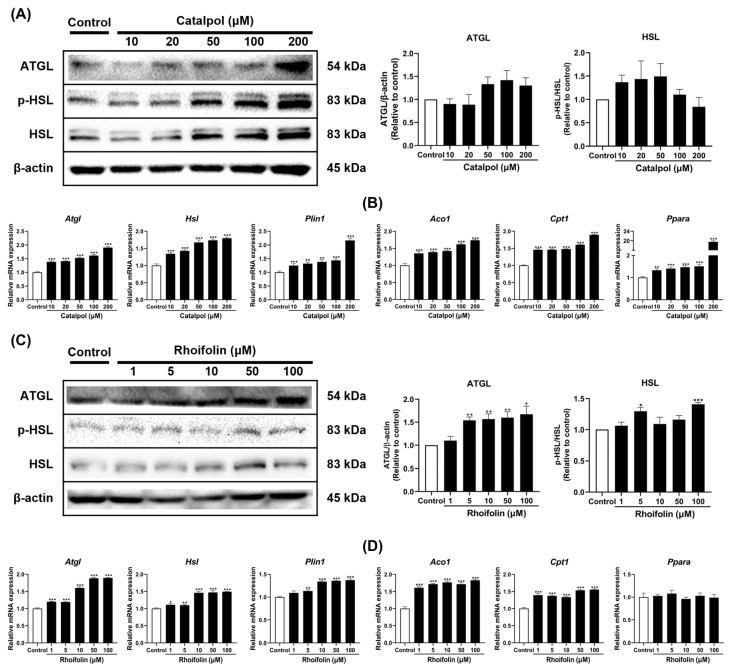
Effects of catalpol (**A**,**B**) and rhoifolin (**C**,**D**) on the expression of lipolytic and β-oxidation markers in 3T3-L1 adipocytes. Target gene mRNA expression was analyzed using qRT-PCR and normalized to *Gapdh* using the 2^−ΔΔCt^ method (*n* = 6). *Gapdh* was used as the housekeeping gene and β-actin as the loading control (*n* = 3). Statistical analysis was performed using Student’s *t*-test for comparisons between the control and each treatment group. Data are presented as mean ± standard error of the mean (SEM). * *p* < 0.05, ** *p* < 0.01, and *** *p* < 0.001 vs. control.

Lipolysis and fatty acid oxidation were evaluated to determine whether catalpol and rhoifolin stimulate lipid catabolism in 3T3-L1 adipocytes. Both catalpol and rhoifolin significantly enhanced the expression of lipolysis-related genes, including *Atgl*, *Hsl*, and *Plin1*, with corresponding increases in protein levels (Figure 6A,C). Additionally, catalpol dose-dependently increased the expression of fatty acid oxidation genes, such as *Aco1*, *Cpt1*, and *Ppara* (Figure 6B), whereas rhoifolin did not significantly alter *Ppara* expression (Figure 6D). Overall, these findings highlight that catalpol and rhoifolin regulate lipid metabolism and catabolism, thereby promoting the fat-browning in 3T3-L1 adipocytes.

### 2.6. Effects of Catalpol and Rhoifolin on UCP1 Through Activation of β3-AR Signaling Pathway in 3T3-L1 Adipocytes

**Figure 7:**
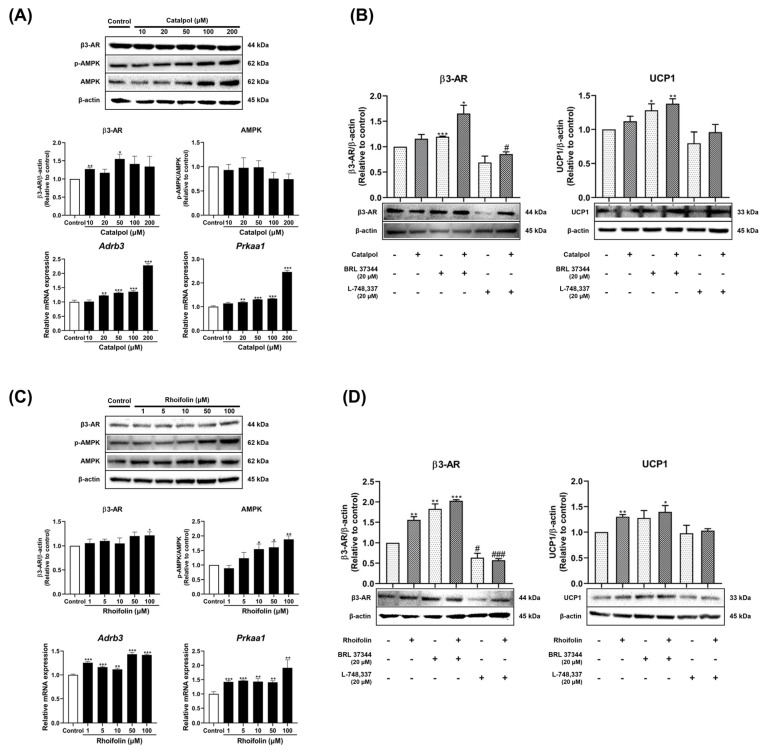
Effects of catalpol (**A**,**B**) and rhoifolin (**C**,**D**) on the expression of fat browning-associated β3-AR signaling pathway in 3T3-L1 adipocytes. Target gene mRNA expression was analyzed using qRT-PCR and normalized to *Gapdh* using the 2^−ΔΔCt^ method (*n* = 6). *Gapdh* was used as the housekeeping gene for mRNA analysis, and β-actin as the loading control for protein analysis (*n* = 3). The control group represents cells treated with vehicle (0.5% DMSO). Co-treatment with β3-AR agonist (BRL37344, 20 µM) and active compounds further increases the expression of β3-AR and UCP1. In addition, Blockade of β3-AR by antagonist (L-748,337, 20 µM) reduces the expression of UCP1 and abolishes the increase in β3-AR and UCP1 stimulated by catalpol (200 µM) and rhoifolin (100 µM). Statistical analysis was performed using Student’s *t*-test for comparisons between the control and each treatment group. Data are presented as mean ± standard error of the mean (SEM). * *p* < 0.05, ** *p* < 0.01, and *** *p* < 0.001 compared with the control (increase); # *p* < 0.05, and ### *p* < 0.001 compared with the control (decrease).

The expression of β3-AR and the phosphorylation of AMPK were examined to investigate the molecular mechanisms underlying catalpol and rhoifolin in promoting adipocyte browning. Both compounds increased the mRNA expression of both *Adrb3* and *Prkaa1*; however, only rhoifolin significantly elevated the corresponding protein levels (Figure 7A,C). Additionally, further investigations were conducted to clarify the molecular pathways underlying the browning effects of these compounds. During 3T3-L1 adipocyte differentiation, the maximum concentrations of catalpol and rhoifolin were applied independently, alone or in combination with a β3-AR agonist (BRL 37344, 20 µM) or antagonist (L-748,337, 20 µM). Subsequently, protein expression levels of β3-AR and UCP1 were measured to validate the signaling pathway. Pharmacological inhibition of β3-AR with the antagonist reduced basal expression of both β3-AR and UCP1 and effectively abolished the upregulation previously induced by catalpol and rhoifolin (Figure 7B,D). The β3-AR agonist further increased these markers in combination with the highest doses, indicating that catalpol and rhoifolin promote fat browning specifically via a β3-AR-dependent UCP1 activation pathway.

## 3. Discussion

Inducing adipocyte browning represents a promising therapeutic strategy for obesity. In this study, RGE, LJE, and their active compounds, catalpol and rhoifolin, were identified as effective botanical agents for promoting this process. Considering the established physiological benefits of YST in traditional medicine, its potential to induce a metabolic shift in 3T3-L1 adipocytes was investigated. These findings indicate that these herbal extracts and compounds significantly suppress lipid accumulation and reduce adipocyte hypertrophy. The lipid droplets observed in Figure 2B appear reduced in size and number, a morphological feature often associated with browning. Additionally, our molecular evidence, specifically the upregulation of UCP1 and increased expression of mitochondrial biogenesis markers, provides strong support for browning-like metabolic remodeling. However, the lack of quantitative analysis regarding droplet size and density suggests that these morphological observations should be interpreted with caution. Furthermore, as 7 days of differentiation may reflect an early or intermediate state of adipocyte maturation, and cell viability was assessed for only 72 h, the observed reduction in lipid accumulation may involve a combination of mechanisms. These include the inhibition of adipogenesis, increased lipolysis, induction of browning, or mild cytotoxic effects at higher concentrations. To better distinguish these processes and ensure that the observed metabolic shift is not an effect of long-term cellular stress, further studies should integrate viability and differentiation assays over the full treatment period and incorporate later time points (e.g., days 10–12).

PGC-1α, a key transcriptional coactivator, regulates energy metabolism, mitochondrial biogenesis, and fatty acid oxidation across various tissues [34]. In brown and beige adipocytes, PGC-1α promotes thermogenesis by inducing mitochondrial genes, such as *Ucp1*, *Nrf1*, and *Tfam* [35]. These results confirmed that treatment with the active compounds significantly upregulates these targets, indicating enhanced thermogenic activity and promotion of adipocyte browning. To verify adipocyte browning with greater precision, the expression of beige adipocyte-specific markers was evaluated. Common markers used to identify beige adipocytes include *Cd137*, *Cited*, *Tbx1*, *Cidea*, *Fgf21*, and *Tmem26*. Evaluating the expression profiles of multiple markers provides a more robust approach for determining the successful induction of adipocyte browning than relying on a single gene [36,37]. In this study, treatment with RGE, LJE, and their active compounds, catalpol and rhoifolin, significantly increased the expression of these markers. These findings suggest that the active compounds increase thermogenic marker expression and promote the phenotypic transition of white adipocytes to a beige adipocyte-like state.

As previously described, the adipocyte browning involves significant mitochondrial remodeling and increased biogenesis. This transition is central to thermogenic activity; compared to white adipocytes, beige adipocytes exhibit higher mitochondrial density and elevated levels of oxidative phosphorylation complexes, facilitate energy expenditure by converting stored triglycerides into heat [38]. In this study, treatment with RGE, LJE, and their active compounds, catalpol and rhoifolin, increased the expression of thermogenic markers, such as UCP1 and PGC-1α, and significantly upregulated the genes involved in mitochondrial biogenesis, including *Nrf1*, *Tfam*, and *Cox4*. Upregulation of *Nrf1* and *Tfam* promotes mitochondrial DNA replication and protein synthesis, while *Cox4* serves as a key indicator of mitochondrial functional activity [39,40]. The concurrent increase in these markers indicates robust induction of mitochondrial biogenesis and functional activation. Immunofluorescence staining further confirmed these findings by demonstrating increased protein levels consistent with the mRNA expression data. Collectively, these findings suggest that RGE, LJE and their active compounds, catalpol and rhoifolin, enhance the thermogenic pathway by promoting both mitochondrial biogenesis and functional maturation.

The browning of adipocytes enhances thermogenic activity and regulates lipid metabolism by inhibiting lipid accumulation and promoting energy expenditure [41]. To investigate this mechanism, the expression of key regulators of adipogenesis and lipogenesis was analyzed. Adipogenesis describes the differentiation of preadipocytes into mature adipocytes, with PPARγ and C/EBPα serving as key transcriptional regulators [42]. The results showed a significant increase in C/EBPα expression, indicating successful differentiation into a mature and functional adipocyte phenotype. Additionally, the increased expression of PPARγ and C/EBPα observed in this study, when considered alongside the concurrent upregulation of key thermogenic regulators such as UCP1 and PGC-1α, indicates that these cells are being metabolically reprogrammed into a thermogenic, oxidative state rather than simply accumulating more lipids. In contrast, lipogenesis involves fatty acid synthesis and triglyceride formation in differentiated adipocytes, with enzymes such as FASN and ACC playing critical roles [43]. In this study, a significant reduction was observed in *Fasn* mRNA expression. These findings suggest that, although the differentiation of preadipocytes toward a beige phenotype was increased, fatty acid synthesis and subsequent triglyceride accumulation decreased concurrently. This finding is consistent with those of previous studies by Jung et al. [44], which reported similar metabolic shifts. Consequently, catalpol and rhoifolin reduced energy storage and promoted metabolic reprogramming toward energy expenditure, consistent with the previously confirmed mitochondrial activation and adipocyte browning pathways.

Lipolysis in adipocytes, the sequential hydrolysis of triglycerides into free fatty acids and glycerol, is primarily regulated by adipose triglyceride lipase (ATGL), hormone-sensitive lipase (HSL), and perilipin1 (PLIN1) [45]. ATGL initiates this process by hydrolyzing triglycerides into diacylglycerols, followed by HSL-mediated conversion of diacylglycerols into monoacylglycerols. PLIN1, a lipid droplet-associated protein, acts as a regulatory barrier controlling lipase access to stored triglycerides. Protein kinase A (PKA)-dependent phosphorylation induces a conformational change in PLIN1 that increases lipase accessibility and facilitates the lipolytic activity of ATGL and HSL [46]. In this study, treatment with catalpol and rhoifolin significantly upregulated the mRNA expression of *Atgl*, *Hsl*, and *Plin1*. Rhoifolin treatment significantly increased the protein level of ATGL and phosphorylation of HSL. Along with the previously observed mitochondrial biogenesis and functional activation, this lipolysis stimulation potentially provides the substrates required for thermogenesis and supports adipocyte browning. Lipolysis is closely associated with β-oxidation because free fatty acids released during lipolysis undergo β-oxidation after entering the mitochondria [47]. In this pathway, ACO1 serves as the initiating enzyme of β-oxidation, catalyzing the initial oxidation of long-chain fatty acids [48]. Additionally, CPT1 facilitates mitochondrial uptake of long-chain fatty acids by conjugating with carnitine, enabling their transport across the mitochondrial membrane [49]. PPARα acts as a central transcription factor coordinating the expression of genes involved in fatty acid oxidation [50]. Consistent with these established mechanisms, the results showed that treatment with catalpol and rhoifolin significantly upregulated the mRNA expression of three key targets: *Aco1*, *Cpt1*, and *Ppara*. This upregulation suggests efficient mitochondrial oxidation of fatty acids released during lipolysis. Induction of these oxidative markers indicates that the active compounds effectively accelerate lipid metabolism by promoting the transition from lipid breakdown to energy utilization. Collectively, these results imply that enhanced mitochondrial biogenesis, lipolysis, and fatty acid oxidation collectively contribute to the promotion of a browning-like phenotype. Although these results consistently demonstrate the browning-like metabolic remodeling, the use of a single point limits our ability to resolve the temporal hierarchy between these processes. Therefore, further studies tracking these changes over multiple time points are required to clarify whether browning precedes or concurrently results from increased lipolysis and inhibited lipogenesis.

The stimulation of lipid metabolism observed alongside adipocyte browning in this study may be attributed to the activation of the β3-AR signaling pathway. Specifically, β3-AR activation stimulates AMPK, a central kinase regulating energy homeostasis via cAMP-dependent signaling and inhibits lipogenesis through the phosphorylation of ACC [51]. This signaling pathway further supports mitochondrial activation and thermogenesis by modulating the PGC-1α–UCP1 axis [52]. Concurrent β3-AR activation promotes lipolysis by inducing PKA-mediated phosphorylation and activation of PLIN and HSL [53]. The consistent upregulation observed in our dataset strongly suggests that the adipocyte browning response effect is driven by the β3-AR signaling pathway. The rhoifolin-induced increase in UCP1 expression was robustly supported by the outcomes of β3-AR agonist and antagonist treatments. Compared to the outcomes of AMPK activator and inhibitor treatments (Figure S4), the β3-AR-related markers exhibited a more pronounced trend, further supporting that the adipocyte browning effects of catalpol and rhoifolin are primarily mediated through the β3-AR signaling pathway.

Despite the significant findings, some limitations warrant attention in future studies. First, slight discrepancies were observed in the expression trends of certain molecular targets, which may be attributed to the distinct temporal expression profiles of each target or the selective modulation of specific pathways by the active compounds. Second, observations were confined primarily to the post-differentiation phase. To strengthen the evidence for the anti-obesity effects of catalpol and rhoifolin, investigating cellular dynamics beyond the completion of differentiation into the mature adipocytes is necessary. Third, given that this study relied solely on the 3T3-L1 cell line, further research using diverse models, such as primary preadipocytes from patients with obesity or mesenchymal stem cells, is required to confirm the generalizability of these pro-browning effects. Finally, these in vitro findings require validation in vivo. The evaluation of energy expenditure, body weight, and glucose homeostasis will supplement current whole-body metabolic data, while measurements of functional thermogenesis can be verified through oxygen consumption rate and proton leakage. Subsequent investigations in animal models are essential to determine whether optimal doses of these compounds can effectively induce adipocyte browning and produce systemic anti-obesity effects within a complex physiological environment.

## 4. Materials and Methods

### 4.1. Chemicals and Reagents

Dried roots of *R*. *glutinosa* (1.0 kg) and dried aerial parts of *L*. *japonica* (1.0 kg) were procured from the Oriental Medicine Pharmacy at Dongguk University Hospital in July 2024. Voucher specimens of *R. glutinosa* (DGU-917) and *L. japonica* (DGU-918) were authenticated and deposited at the Integrated Research Institute for Drug Development, Dongguk University, in July 2024. Each plant material was mechanically pulverized into a fine powder and subjected to ultrasonication-assisted extraction using 70% aqueous ethanol (10 L × 3 times, 99 min per cycle) at ambient temperature. The combined extracts were initially filtered through cotton wool and subsequently concentrated under reduced pressure at 40 °C utilizing a rotary vacuum evaporator. Prior to subsequent chemical analyses, the sample solutions were passed through a 0.22 μm syringe filter. This procedure afforded the crude extracts targeting catalpol and rhoifolin, yielding RGE (254.0 g) and LJE (162.0 g), which correspond to extraction yields of 25.4% and 16.2%, respectively. The ultrasound-assisted extraction protocols for both medicinal plants were performed in accordance with previously reported methods [54,55]. Catalpol and rhoifolin were obtained from ChemFaces Biochemical (Wuhan, China). 3T3-L1 preadipocytes were obtained from the Korean Cell Line Bank (Seoul, Republic of Korea). Dulbecco’s modified Eagle’s medium/high glucose (DMEM), DAPI, fetal bovine serum (FBS), newborn bovine calf serum (NCS), and penicillin–streptomycin (P–S) were purchased from Thermo Fisher Scientific (Waltham, MA, USA). Sigma-Aldrich (St. Louis, MO, USA) supplied dexamethasone (DEX), insulin, 5-amino-imidazole-4-carboxamide ribonucleotide (AICAR), phosphate-buffered saline (PBS), 10% neutral buffered formalin, Oil Red O, and dorsomorphin. Dimethyl sulfoxide (DMSO) and MTT were sourced from Glentham Life Sciences (Corsham, UK). Isopropanol, 3-isobutyl-1-methylxanthine (IBMX), goat anti-rabbit IgG, and goat anti-mouse IgG secondary antibodies were obtained from Merck (Union County, NJ, USA). Primary antibodies against ATGL (Cat #2439, 1:1000), PKA (5842, 1:1000), PLIN (9349, 1:1000), PPARγ (2435, 1:1000), C/EBPα (8178, 1:1000), HSL (18381, 1:1000), phospho-HSL (p-HSL; 4139, 1:1000), AMPK (2532, 1:1000), phospho-AMPK (p-AMPK; 2531, 1:500), ACC (3662, 1:1000), phospho-ACC (p-ACC; 3661, 1:500) and β-actin (4967, 1:1000) were purchased from Cell Signaling Technology (Danvers, MA, USA). Antibodies against UCP1 (sc-293418, 1:500), PGC-1α (sc-518025, 1:500) and β3-AR (sc-515763, 1:500) were sourced from Santa Cruz Biotechnology (Santa Cruz, CA, USA). Immun-Blot^®^ PVDF membranes, phosphatase and protease inhibitor cocktails, skim milk, 10% Tween-20, 2-mercaptoethanol (2-βME), 4 × Laemmli sample buffer, and iQ™ SYBR^®^ Green Supermix were obtained from Bio-Rad (Hercules, CA, USA). PCR-grade RNase-free water was obtained from Jena Bioscience (Munich, Germany). BRL-37344 and L-748,337 were sourced from Tocris Bioscience (Bristol, UK). NucleoZOL reagent was supplied by Macherey-Nagel (Düren, Germany), and complementary DNA (cDNA) synthesis was performed with the ReverTra Ace^®^ qPCR RT Kit (TOYOBO, Osaka, Japan).

### 4.2. Cell Culture and Differentiation

To initiate cell culture, 3T3-L1 preadipocytes were seeded and incubated for 48 h in DMEM supplemented with 10% NCS and 1% P–S at 37 °C in a 5% CO_2_ incubator. Growth arrest was achieved by continuing incubation in the same medium until the cells reached confluence. When the cells reached full confluence, differentiation was initiated by transitioning to induction medium consisting of DMEM supplemented with 10% FBS, 0.5 mM IBMX, 1 μM DEX, and 10 μg/mL insulin. After 3 days of induction, the medium was replaced with progression medium containing DMEM, 10% FBS, and insulin (10 μg/mL), and the cells were maintained for an additional 2 days. During differentiation, cells were exposed to graded doses of individual herbal extracts (1–100 μg/mL) or their bioactive compounds (1–200 μM) (Figure S5). To investigate pathways involved in adipocyte browning, additional treatments included AMPK modulators—AICAR (10 μM) as an activator and dorsomorphin (5 μM) as an inhibitor—as well as β3-adrenergic receptor agents, including the agonist BRL-37344 (20 μM) and the antagonist L-748,337 (20 μM). These agents were administered alone or combined with the herbal treatments during differentiation. All experiments, except for the MTT assay, were performed 7 days after differentiation.

### 4.3. Cell Viability Assay

Cell viability was assessed using the MTT assay, a colorimetric method based on tetrazolium reduction. The assay relies on mitochondrial enzymes, including NADPH-dependent oxidoreductases and dehydrogenases, which convert yellow tetrazolium salts into insoluble purple formazan crystals in viable cells [56,57]. 3T3-L1 preadipocytes were seeded at 1 × 10^4^ cells per well in 96-well plates and incubated overnight in DMEM supplemented with 10% NCS and 1% P–S. Once cells reached ~80–90% confluence, they were treated with RGE and LJE (1, 5, 10, 25, 50, 75, and 100 μg/mL) or their active compounds, catalpol (5, 10, 20, 50, 100, 200, and 400 μM) and rhoifolin (0.5, 1, 5, 10, 50, 100, and 200 μM). Treatments were administered for 24, 48, and 72 h, with 0.5% DMSO as the vehicle control. At the end of each period, 20 μL of MTT solution (5 mg/mL) was added to each well, followed by a 2 h incubation. The supernatant was then discarded, and 100 μL of DMSO was added to dissolve the formazan crystals. After 10 min, absorbance at 540 nm was measured using an xMark™ microplate spectrophotometer (Bio-Rad, Hercules, CA, USA). Cell viability was expressed as a percentage of the control and reported as the mean of six independent experiments.

### 4.4. Oil Red O Staining

Intracellular lipid accumulation was evaluated using Oil Red O staining of differentiated 3T3-L1 adipocytes. Cells were seeded in 24-well plates at 1 × 10^5^ cells per well and allowed to differentiate. After differentiation, cells were rinsed twice with PBS and fixed with 10% formalin for 30 min at room temperature (25 °C). After fixation, residual formalin was removed by washing cells twice with deionized water (DW) to eliminate excess fixative. Cells were then incubated with a freshly prepared Oil Red O working solution for 30 min at room temperature to stain neutral lipids. Excess dye was discarded, and the cells were rinsed three times with DW to remove unbound dye. Oil Red O stained lipid droplets were visualized under a microscope to confirm differentiation and lipid storage. For quantification of lipid accumulation, 100% isopropanol was added to each well to elute the dye, followed by gentle agitation for 30 min at room temperature. The extracted dye was transferred to 96-well plates, and absorbance was measured at 520 nm using a Bio-Rad microplate spectrophotometer (Bio-Rad, Hercules, CA, USA).

### 4.5. Western Blot Analysis

For immunoblotting, 3T3-L1 adipocytes were cultured in 6-well plates at 1 × 10^6^ cells per well. After incubation, cells were rinsed with ice-cold PBS and collected into prechilled 1.5 mL microcentrifuge tubes. Cells were then centrifuged at 14,000 rpm for 5 min at 4 °C, and the supernatant was removed. Cell pellets were lysed on ice for 30 min in 100 μL of RIPA buffer containing protease and phosphatase inhibitors (98:1:1). Lysates were clarified through centrifugation at 14,000× *g* for 20 min at 4 °C, and protein concentrations were determined using the Bradford assay. For each sample, 40 μg of total protein was mixed with sample buffer and distilled water to 20 μL. Mixtures were heated at 100 °C for 10 min and briefly centrifuged at 14,000× *g* for 3 min to remove insoluble debris. Proteins were separated through 10% SDS-polyacrylamide gel electrophoresis and transferred onto PVDF membranes (Immun-Blot^®^) for 1 h. Membranes were blocked in 5% skim milk diluted in Tris-buffered saline with 0.1% Tween-20 (TBS-T) for 1 h at room temperature with gentle shaking. After blocking, they were washed five times for 5 min each in TBS-T and incubated overnight at 4 °C with primary antibodies diluted in TBS-T. The next day, membranes were washed three times and incubated for 1 h at room temperature with horseradish peroxidase-conjugated secondary antibodies (goat anti-rabbit or goat anti-mouse, 1:4000 in TBS-T with 5% skim milk). Bands were visualized using a Bio-Rad ChemiDoc XRS+ imaging system, and intensities were analyzed with Image Lab Software 3.0 (Bio-Rad).

### 4.6. Quantitative Real-Time Polymerase Chain Reaction

For RNA extraction, differentiated 3T3-L1 adipocytes were scraped from 6-well plates and suspended in 500 μL of NucleoZOL reagent. An equal volume of RNase-free PCR-grade water was added to promote phase separation, and the mixture was vortexed briefly before centrifugation at 12,000× *g* for 15 min. The aqueous supernatant (1 mL) was collected and mixed with 1 mL isopropanol to precipitate RNA. After incubating at room temperature for 10 min, tubes were centrifuged again at 12,000× *g* for 10 min to collect RNA pellets. The pellets were rinsed with 500 μL 75% ethanol and centrifuged at 8000× *g* for 3 min. Pellets were then air-dried after discarding the supernatant and resuspended in RNase-free water. RNA quantity and quality were measured using a NanoDrop spectrophotometer (Thermo Scientific, Wilmington, DE, USA). To obtain purified messenger RNA, the total RNA was further processed using the NucleoTrap^®^ mRNA Mini purification kit. cDNA was synthesized from the isolated mRNA using the ReverTra Ace^®^ qPCR RT kit, following the instructions of the manufacturer. Real-time PCR was performed with iQ™ SYBR^®^ Green Supermix on a CFX384 Touch Real-Time PCR Detection System (Bio-Rad, Hercules, CA, USA). Relative gene expression was normalized to GAPDH levels using the 2^−ΔΔCt^ method. Table 1 lists the primer sequences used for amplification.

### 4.7. Immunofluorescence

Immunofluorescence staining was performed on 3T3-L1 adipocytes to investigate the effects of individual herbal extracts and their active compounds on UCP1 expression and mitochondrial biogenesis. Cells were seeded and differentiated for 7 days on sterilized glass coverslips placed in a 24-well plate. Mitochondria were stained by incubating the cells for 30 min in medium containing 50 nM MitoTracker^®^ Red CMXRos, followed by three washes with PBS. Cells were then fixed with 10% formalin for 15 min at room temperature and washed three times with PBS. For blocking and permeabilization, cells were incubated in blocking buffer containing 5% bovine serum albumin and 0.1% Triton^®^ X-100 in PBS for 1 h at 4 °C. After washing with the same buffer, the cells were incubated overnight at 4 °C with a FITC-conjugated primary antibody against UCP1 (1:500). Following antibody incubation, the cells were rinsed three times with PBS and treated with DAPI (10 μg/mL, 1:1000) for 1 min to visualize nuclei, then washed again with PBS. Fluorescent images were acquired using a Nikon C1 confocal laser scanning microscope and processed with EZ-C1 software 3.9 (Nikon, Tokyo, Japan). Fluorescence intensity was quantified using ImageJ software (v1.54; National Institutes of Health, Bethesda, MD, USA) [58].

### 4.8. Statistical Analysis

All results are presented as mean ± SEM from six independent replicates, except for Western blot and immunofluorescence analyses, which were performed in triplicate. Statistical comparisons between control and treatment groups were conducted using Student’s *t*-test, and a *p* < 0.05 was considered statistically significant.

## 5. Conclusions

This study demonstrates that catalpol and rhoifolin, bioactive compounds derived from *R. glutinosa* and *L. japonica*, promote adipocyte browning and metabolic activity in 3T3-L1 cells. Both compounds significantly reduced lipid accumulation and upregulated the expression of UCP1 and PGC-1α, key markers of thermogenic activation. Furthermore, the observed increase in ATGL and HSL phosphorylation indicates a coordinated activation of lipolysis. These metabolic adaptations are primarily mediated through the β3-AR signaling pathway. Collectively, these findings establish catalpol and rhoifolin as potent inducers of adipocyte browning and energy expenditure, highlighting their potential as evidence-based natural agents for obesity management, although further validation in vivo and in clinical studies is required.

## Figures and Tables

**Table 1:** The primer sequence is used for quantitative RT-PCR.

Gene	Forward	Reverse
*Acaca*	GGGAACATCCCCACGCTAAA	GAAAGAGACCATTCCGCCCA
*Aco1*	ATCCAGACTTCCAACATFAG	AACCACATGATTTCTTCAGG
*Adrb3*	TTGTCCTGGTGTGGATCGTG	TTGGAGGCAAAGGAACAGCA
*Atgl*	TTCACCATCCGCTTGTTGGAG	AGATGGTCACCCAATTTCCTC
*Cd137*	GGTCTGTGCTTAAGACCGGG	TCTTAATAGCTGGTCCTCCCTC
*Cebpa*	AGGTGCTGGAGTTGACCAGT	CAGCCTAGAGATCCAGCGAC
*Cidea*	CGGGAATAGCCAGAGTCACC	TGTGCATCGGATGTCGTAGG
*Cited*	AACCTTGGAGTGAAGGATCGC	GTAGGAGAGCCTATTGGAGATGT
*Cox4*	TGACGGCCTTGGACGG	CGATCAGCGTAAGTGGGGA
*Cpt1*	GTGTTGGAGGTGACAGACTT	CACTTTCTCTTTCCACAAGG
*Fasn*	TTGCTGGCACTACAGAATGC	AACAGCCTCAGAGCGACAAT
*Fgf21*	CGTCTGCCTCAGAAGGACTC	TCTACCATGCTCAGGGGGTC
*Hsl*	GCACTGTGACCTGCTTGGT	CTGGCACCCTCACTCCATA
*Lpl*	AGGACCCCTGAAGACACAGCT	TGTACAGGGCGGCCACAAGT
*Nrf1*	GCTAATGGCCTGGTCCAGAT	CTGCGCTGTCCGATATCCTG
*Pgc* *-* *1a*	ATGTGCAGCCAAGACTCTGTA	CGCTACACCACTTCAATCCAC
*Plin1*	GCAAGAAGAGCTGAGCAGAC	AATCTGCCCACGAGAAAGGA
*Ppara*	GAGAGGGCACACGCTAGGAA	GAACACCAATGTTCGGAGCC
*Pparg*	CAAGAATACCAAAGTGCGATCAA	GAGCTGGGTCTTTTCAGAATAATAAG
*Prdm16*	GATGGGAGATGCTGACGGAT	TGATCTGACACATGGCGAGG
*Prkaa1*	GCGCCATGCGCAGACTCA	GTGTCCCCCAGGATGTAGTGG
*Srebf1*	GCTTAGCCTCTACACCAACTGGC	ACAGACTGGTACGGGCCACAAG
*Tbx1*	AGCGAGGCGGAAGGGA	CCTGGTGACTGTGCTGAAGT
*Tfam*	ATGTGGAGCGTGCTAAAAGC	GGATAGCTACCCATGCTGGAA
*Tmem26*	CCATGGAAACCAGTATTGCAGC	ATTGGTGGCTCTGTGGGATG
*Ucp1*	CCTGCCTCTCTCGGAAACAA	GTAGCGGGGTTTGATCCCAT
*G* *apdh*	TTGTTGCCATCAACGACCCC	GCCGTTGAATTTGCCGTGAG

## Data Availability

The data presented in this research are available from the corresponding authors upon request.

## Supplementary Materials

The following supporting information can be downloaded at https://www.mdpi.com/article/10.3390/ph19050787/s1: Figure S1: Effects of Extracts of *Rehmannia glutinosa* (RGE) and *Lonicera japonica* (LJE) on cell viability and lipid accumulation in 3T3-L1 adipocytes; Figure S2: Effects of RGE (A, B) and LJE (C, D) on the expression of thermogenic and beige fat-specific markers in 3T3-L1 adipocytes; Figure S3: Effects of RGE and LJE on mitochondrial biogenesis in 3T3-L1 adipocytes; Figure S4: Effects of catalpol and rhoifolin on the expression of fat browning through AMPK signaling pathway in 3T3-L1 adipocytes. Figure S5: 3T3-L1 cell differentiation process.

## Abbreviations

The following abbreviations are used in this manuscript:

2-βME	2-mercaptoethanol
ACC	Acetyl-CoA carboxylase
ACO1	Acyl-CoA carboxylase 1
AMPK	Adenosine monophosphate–activated protein kinase
ATGL	Adipose triglyceride lipase
BAT	Brown adipose tissue
C/EBP	CCAAT/enhancer binding protein
CD137	Tumor necrosis factor receptor superfamily, member 9
CIDEA	Cell death-inducing DNA fragmentation factor alpha-like effector A
CITED	Cbp/p300-interacting transactivator, with Glu/Asp-rich carboxy-terminal domain
COX4	Cytochrome c oxidase subunit 4
CPT1	Carnitine palmitoyltransferase 1
Ct	Cycle threshold
DAPI	4′,6-Diamidino-2-phenylindole
DEX	Dexamethasone
DMEM	Dulbecco’s modified eagle’s medium/high glucose
DMSO	Dimethyl sulfoxide
DW	Deionized water
FASN	Fatty acid synthase
FBS	Fetal bovine serum
FITC	Fluorescein isothiocyanate
GAPDH	Glyceraldehyde-3-phosphate dehydrogenase
HSL	Hormone-sensitive lipase
IBMX	3-isobutyl-1-methylxanthine
LJE	Extract of *Lonicera japonica*
LPL	Lipoprotein lipase
MTT	3-(4,5-dimethylthiazol-2-yl)-2,5-diphenyltetrazolium
NCS	Newborn bovine calf serum
NRF1	Nuclear respiratory factor 1
PBS	Phosphate-buffered saline
PGC-1α	Peroxisome proliferator-activated receptor gamma co-activator 1α
P-S	Penicillin-streptomycin
PKA	Protein kinase A
PLIN1	Perilipin1
PPAR	Peroxisome proliferator-activated receptor
PRDM16	PR domain containing 16
qRT-PCR	Quantitative real-time polymerase chain reaction
RGE	Extract of *Rehmannia glutinosa*
SEM	Standard error of the mean
TBX1	T-box 1
TBS-T	Tris-buffered saline with 0.1% Tween-20
TFAM	Mitochondrial transcription factor A
TMEM26	Transmembrane Protein 26
UCP1	Uncoupling protein 1
USA	United States of America
WAT	White adipose tissue
YST	*Yanggyeoksanhwa-tang*
β3-AR	β3 adrenergic receptor

## References

[1] Fox A., Feng W., Asal V. (2019). What Is Driving Global Obesity Trends? Globalization or “Modernization”?. Glob. Health.

[2] Adolph T.E., Tilg H. (2024). Western Diets and Chronic Diseases. Nat. Med..

[3] Chaney A. (2021). Obesity and Nonalcoholic Fatty Liver Disease. Nurs. Clin..

[4] Bray G.A., Frühbeck G., Ryan D.H., Wilding J.P.H. (2016). Management of Obesity. Lancet.

[5] Müller T.D., Blüher M., Tschöp M.H., DiMarchi R.D. (2022). Anti-Obesity Drug Discovery: Advances and Challenges. Nat. Rev. Drug Discov..

[6] Tak Y.J., Lee S.Y. (2021). Anti-Obesity Drugs: Long-Term Efficacy and Safety: An Updated Review. World J. Mens Health.

[7] Machado S.A., Pasquarelli-do-Nascimento G., da Silva D.S., Farias G.R., de Oliveira Santos I., Baptista L.B., Magalhães K.G. (2022). Browning of the White Adipose Tissue Regulation: New Insights into Nutritional and Metabolic Relevance in Health and Diseases. Nutr. Metab..

[8] Wang W., Seale P. (2016). Control of Brown and Beige Fat Development. Nat. Rev. Mol. Cell Biol..

[9] Richard A.J., White U., Elks C.M., Stephens J.M., Feingold K.R., Ahmed S.F., Anawalt B., Blackman M.R., Boyce A., Chrousos G., Corpas E., de Herder W.W., Dhatariya K., Dungan K. (2000). Adipose Tissue: Physiology to Metabolic Dysfunction. Endotext.

[10] Jones S.A., Ruprecht J.J., Crichton P.G., Kunji E.R.S. (2024). Structural Mechanisms of Mitochondrial Uncoupling Protein 1 Regulation in Thermogenesis. Trends Biochem. Sci..

[11] Rayalam S., Yang J.-Y., Ambati S., Della-Fera M.A., Baile C.A. (2008). Resveratrol Induces Apoptosis and Inhibits Adipogenesis in 3T3-L1 Adipocytes. Phytother. Res. PTR.

[12] Baboota R.K., Singh D.P., Sarma S.M., Kaur J., Sandhir R., Boparai R.K., Kondepudi K.K., Bishnoi M. (2014). Capsaicin Induces “Brite” Phenotype in Differentiating 3T3-L1 Preadipocytes. PLoS ONE.

[13] Song Z., Revelo X., Shao W., Tian L., Zeng K., Lei H., Sun H.-S., Woo M., Winer D., Jin T. (2018). Dietary Curcumin Intervention Targets Mouse White Adipose Tissue Inflammation and Brown Adipose Tissue UCP1 Expression. Obesity.

[14] Choi S.M., Lee H.S., Lim S.H., Choi G., Choi C.-I. (2024). Hederagenin from *Hedera helix* Promotes Fat Browning in 3T3-L1 Adipocytes. Plants.

[15] Choi S.M., Lim S.H., Lee H.S., Choi G., Kim M.J., Kim H., Choi C.-I. (2025). Coixol and Sinigrin from *Coix lacryma-jobi* L. and *Raphanus sativus* L. Promote Fat Browning in 3T3-L1 Adipocytes. Pharmaceuticals.

[16] Tak M.-J., Tark M.-R., Kang K.-H., Ko W.-S., Yoon H.-J. (2010). The Inhibitory Effects of Yang Geouk San Hwa-Tang on LPS-stimulated inflammation in RAW264.7 macrophage cells. J. Korean Med. Ophthalmol. Otolaryngol. Dermatol..

[17] Hotamisligil G.S. (2017). Inflammation, Metaflammation and Immunometabolic Disorders. Nature.

[18] Bian Z., Zhang R., Zhang X., Zhang J., Xu L., Zhu L., Ma Y., Liu Y. (2023). Extraction, Structure and Bioactivities of Polysaccharides from *Rehmannia glutinosa*: A Review. J. Ethnopharmacol..

[19] Yang X., Yu A., Hu W., Zhang Z., Ruan Y., Kuang H., Wang M. (2023). Extraction, Purification, Structural Characteristics, Health Benefits, and Application of the Polysaccharides from *Lonicera japonica* Thunb.: A Review. Molecules.

[20] Zhang T., Liu H., Bai X., Liu P., Yang Y., Huang J., Zhou L., Min X. (2020). Fractionation and Antioxidant Activities of the Water-Soluble Polysaccharides from *Lonicera japonica* Thunb. Int. J. Biol. Macromol..

[21] Chao J.C.-J., Chiang S.-W., Wang C.-C., Tsai Y.-H., Wu M.-S. (2006). Hot Water-Extracted *Lycium barbarum* and *Rehmannia glutinosa* Inhibit Proliferation and Induce Apoptosis of Hepatocellular Carcinoma Cells. World J. Gastroenterol..

[22] Shang X., Pan H., Li M., Miao X., Ding H. (2011). *Lonicera japonica* Thunb.: Ethnopharmacology, Phytochemistry and Pharmacology of an Important Traditional Chinese Medicine. J. Ethnopharmacol..

[23] Lin L., Wang P., Du Z., Wang W., Cong Q., Zheng C., Jin C., Ding K., Shao C. (2016). Structural Elucidation of a Pectin from Flowers of *Lonicera japonica* and Its Antipancreatic Cancer Activity. Int. J. Biol. Macromol..

[24] Wang D., Zhao X., Liu Y. (2017). Hypoglycemic and Hypolipidemic Effects of a Polysaccharide from Flower Buds of *Lonicera japonica* in Streptozotocin-Induced Diabetic Rats. Int. J. Biol. Macromol..

[25] Liu P., Bai X., Zhang T., Zhou L., Li J., Zhang L. (2019). The Protective Effect of *Lonicera japonica* Polysaccharide on Mice with Depression by Inhibiting NLRP3 Inflammasome. Ann. Transl. Med..

[26] Bi Z., Zhao Y., Hu J., Ding J., Yang P., Liu Y., Lu Y., Jin Y., Tang H., Liu Y. (2022). A Novel Polysaccharide from Lonicerae Japonicae Caulis: Characterization and Effects on the Function of Fibroblast-like Synoviocytes. Carbohydr. Polym..

[27] Gao S., Shan Y., Wang Y., Wang W., Li J., Tan H. (2024). Polysaccharides from *Lonicera japonica* Thunb.: Extraction, Purification, Structural Features and Biological Activities-A Review. Int. J. Biol. Macromol..

[28] Wang D., Du N., Wen L., Zhu H., Liu F., Wang X., Du J., Li S. (2017). An Efficient Method for the Preparative Isolation and Purification of Flavonoid Glycosides and Caffeoylquinic Acid Derivatives from Leaves of *Lonicera japonica* Thunb. Using High Speed Counter-Current Chromatography (HSCCC) and Prep-HPLC Guided by DPPH-HPLC Experiments. Molecules.

[29] Zhang X., Yu X., Sun X., Meng X., Fan J., Zhang F., Zhang Y. (2024). Comparative Study on Chemical Constituents of Different Medicinal Parts of *Lonicera japonica* Thunb. Based on LC-MS Combined with Multivariate Statistical Analysis. Heliyon.

[30] Gandhi G.R., Vasconcelos A.B.S., Wu D.-T., Li H.-B., Antony P.J., Li H., Geng F., Gurgel R.Q., Narain N., Gan R.-Y. (2020). Citrus Flavonoids as Promising Phytochemicals Targeting Diabetes and Related Complications: A Systematic Review of In Vitro and In Vivo Studies. Nutrients.

[31] Phan V.K., Nguyen T.M., Minh C.V., Nguyen H.K., Nguyen H.D., Nguyen P.T., Nguyen X.C., Nguyen H.N., Nguyen X.N., Heyden Y.V. (2010). Two New C-Glucosyl Benzoic Acids and Flavonoids from Mallotus Nanus and Their Antioxidant Activity. Arch. Pharm. Res..

[32] Cheng L., Ren Y., Lin D., Peng S., Zhong B., Ma Z. (2017). The Anti-Inflammatory Properties of *Citrus wilsonii* Tanaka Extract in LPS-Induced RAW 264.7 and Primary Mouse Bone Marrow-Derived Dendritic Cells. Molecules.

[33] Sultana B., Yaqoob S., Zafar Z., Bhatti H.N. (2018). Escalation of Liver Malfunctioning: A Step toward Herbal Awareness. J. Ethnopharmacol..

[34] Lin J., Handschin C., Spiegelman B.M. (2005). Metabolic Control through the PGC-1 Family of Transcription Coactivators. Cell Metab..

[35] Shen S.-H., Singh S.P., Raffaele M., Waldman M., Hochhauser E., Ospino J., Arad M., Peterson S.J. (2022). Adipocyte-Specific Expression of PGC1α Promotes Adipocyte Browning and Alleviates Obesity-Induced Metabolic Dysfunction in an HO-1-Dependent Fashion. Antioxidants.

[36] Pilkington A.-C., Paz H.A., Wankhade U.D. (2021). Beige Adipose Tissue Identification and Marker Specificity-Overview. Front. Endocrinol..

[37] Garcia R.A., Roemmich J.N., Claycombe K.J. (2016). Evaluation of Markers of Beige Adipocytes in White Adipose Tissue of the Mouse. Nutr. Metab..

[38] Cedikova M., Kripnerová M., Dvorakova J., Pitule P., Grundmanova M., Babuska V., Mullerova D., Kuncova J. (2016). Mitochondria in White, Brown, and Beige Adipocytes. Stem Cells Int..

[39] Sun J., Leng P., Li X., Guo Q., Zhao J., Liang Y., Zhang X., Yang X., Li J. (2022). Salvianolic Acid A Promotes Mitochondrial Biogenesis and Mitochondrial Function in 3T3-L1 Adipocytes through Regulation of the AMPK-PGC1α Signalling Pathway. Adipocyte.

[40] Das S., Mukhuty A., Mullen G.P., Rudolph M.C. (2024). Adipocyte Mitochondria: Deciphering Energetic Functions across Fat Depots in Obesity and Type 2 Diabetes. Int. J. Mol. Sci..

[41] Bartelt A., Heeren J. (2014). Adipose Tissue Browning and Metabolic Health. Nat. Rev. Endocrinol..

[42] Lefterova M.I., Lazar M.A. (2009). New Developments in Adipogenesis. Trends Endocrinol. Metab..

[43] Wakil S.J., Abu-Elheiga L.A. (2009). Fatty Acid Metabolism: Target for Metabolic Syndrome. J. Lipid Res..

[44] Jung Y., Park J., Kim H.-L., Sim J.-E., Youn D.-H., Kang J., Lim S., Jeong M.-Y., Yang W.M., Lee S.-G. (2018). Vanillic Acid Attenuates Obesity via Activation of the AMPK Pathway and Thermogenic Factors in Vivo and in Vitro. FASEB J..

[45] Markussen L.K., Rondini E.A., Johansen O.S., Madsen J.G.S., Sustarsic E.G., Marcher A.-B., Hansen J.B., Gerhart-Hines Z., Granneman J.G., Mandrup S. (2022). Lipolysis Regulates Major Transcriptional Programs in Brown Adipocytes. Nat. Commun..

[46] Schweiger M., Schreiber R., Haemmerle G., Lass A., Fledelius C., Jacobsen P., Tornqvist H., Zechner R., Zimmermann R. (2006). Adipose Triglyceride Lipase and Hormone-Sensitive Lipase Are the Major Enzymes in Adipose Tissue Triacylglycerol Catabolism*. J. Biol. Chem..

[47] Houten S.M., Wanders R.J.A. (2010). A General Introduction to the Biochemistry of Mitochondrial Fatty Acid β-Oxidation. J. Inherit. Metab. Dis..

[48] Vamecq J., Andreoletti P., El Kebbaj R., Saih F.-E., Latruffe N., El Kebbaj M.H.S., Lizard G., Nasser B., Cherkaoui-Malki M. (2018). Peroxisomal Acyl-CoA Oxidase Type 1: Anti-Inflammatory and Anti-Aging Properties with a Special Emphasis on Studies with LPS and Argan Oil as a Model Transposable to Aging. Oxid. Med. Cell. Longev..

[49] Schlaepfer I.R., Joshi M. (2020). CPT1A-Mediated Fat Oxidation, Mechanisms, and Therapeutic Potential. Endocrinology.

[50] Kersten S. (2014). Integrated Physiology and Systems Biology of PPARα. Mol. Metab..

[51] Thant M.T., Khine H.E.E., Nealiga J.Q.L., Chatsumpun N., Chaotham C., Sritularak B., Likhitwitayawuid K. (2022). α-Glucosidase Inhibitory Activity and Anti-Adipogenic Effect of Compounds from *Dendrobium delacourii*. Molecules.

[52] Chen J., Liu B., Yao X., Yang X., Sun J., Yi J., Xue F., Zhang J., Shen Y., Chen B. (2025). AMPK/SIRT1/PGC-1α Signaling Pathway: Molecular Mechanisms and Targeted Strategies From Energy Homeostasis Regulation to Disease Therapy. CNS Neurosci. Ther..

[53] Han S.-F., Jiao J., Zhang W., Xu J.-Y., Zhang W., Fu C.-L., Qin L.-Q. (2017). Lipolysis and Thermogenesis in Adipose Tissues as New Potential Mechanisms for Metabolic Benefits of Dietary Fiber. Nutrition.

[54] Wang B., Ma X., Yang X., Yang J., Zhou X., Ding X. (2026). *Rehmannia glutinosa* Polysaccharides: A Review on Structural Features, Pharmacological Potential, and Advanced Delivery Systems. Front. Nutr..

[55] Yu H.-C., Huang S.-M., Lin W.-M., Kuo C.-H., Shieh C.-J. (2019). Comparison of Artificial Neural Networks and Response Surface Methodology towards an Efficient Ultrasound-Assisted Extraction of Chlorogenic Acid from *Lonicera japonica*. Molecules.

[56] Lee K., Seo Y.-J., Song J.-H., Chei S., Lee B.-Y. (2019). Ginsenoside Rg1 Promotes Browning by Inducing UCP1 Expression and Mitochondrial Activity in 3T3-L1 and Subcutaneous White Adipocytes. J. Ginseng Res..

[57] van Meerloo J., Kaspers G.J.L., Cloos J., Cree I.A. (2011). Cell Sensitivity Assays: The MTT Assay. Cancer Cell Culture: Methods and Protocols.

[58] Jensen E.C. (2013). Quantitative Analysis of Histological Staining and Fluorescence Using ImageJ. Anat. Rec..

