# Supplementary material for: Fat Browning Effects of Catalpol and Rhoifolin from Rehmannia glutinosa (Gaertn.) and Lonicera japonica (Thunb.) in 3T3-L1 Adipocytes via the β3-AR Signaling Pathway
Source: Pharmaceuticals (Basel). 2026 May 18;19(5):787. doi: 10.3390/ph19050787 (PMC13209893; doi:10.3390/ph19050787)
Supplement: Supplementary file 1 [file pharmaceuticals-19-00787-s001.zip › pharmaceuticals-4292468-supplementary.pdf]

Figure S1, Supplementary Data

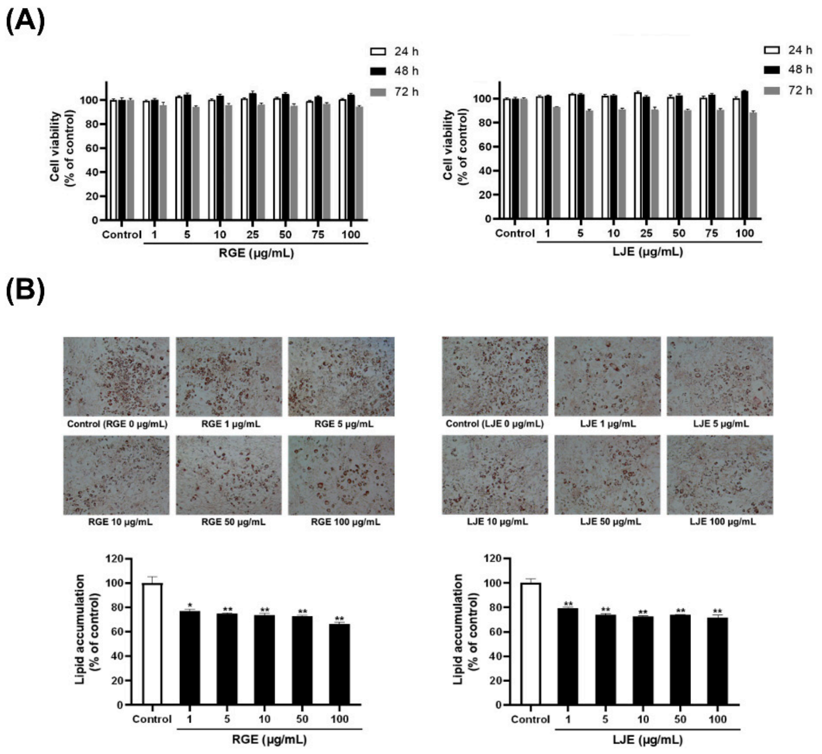

Figure S1. Effects of Extracts of *Rehmannia glutinosa* (RGE) and *Lonicera japonica (*LJE) on cell viability and lipid accumulation in 3T3-L1 adipocytes. (A) Cell viability was assessed after 24 h, 48 h, and 72 h of treatment using the MTT assay. Cells were seeded in 96-well plates and incubated for 24 h prior to treatment. Results are expressed as a percentage of the control (0.5% DMSO) ($n = 6$). Representative cell images were obtained at 100× magnification. (B) Lipid accumulation was evaluated after 7 days of differentiation. Cells were seeded in 24-well plates, with lipid droplets stained with Oil Red O, extracted using isopropanol, and quantified at 520 nm using a microplate reader. Data are expressed as a percentage of the control (0.5% DMSO) and reported as mean ± standard error of the mean (SEM) from triplicates. * $p < 0.05$, and ** $p < 0.01$ vs. control.

Figure S2, Supplementary Data

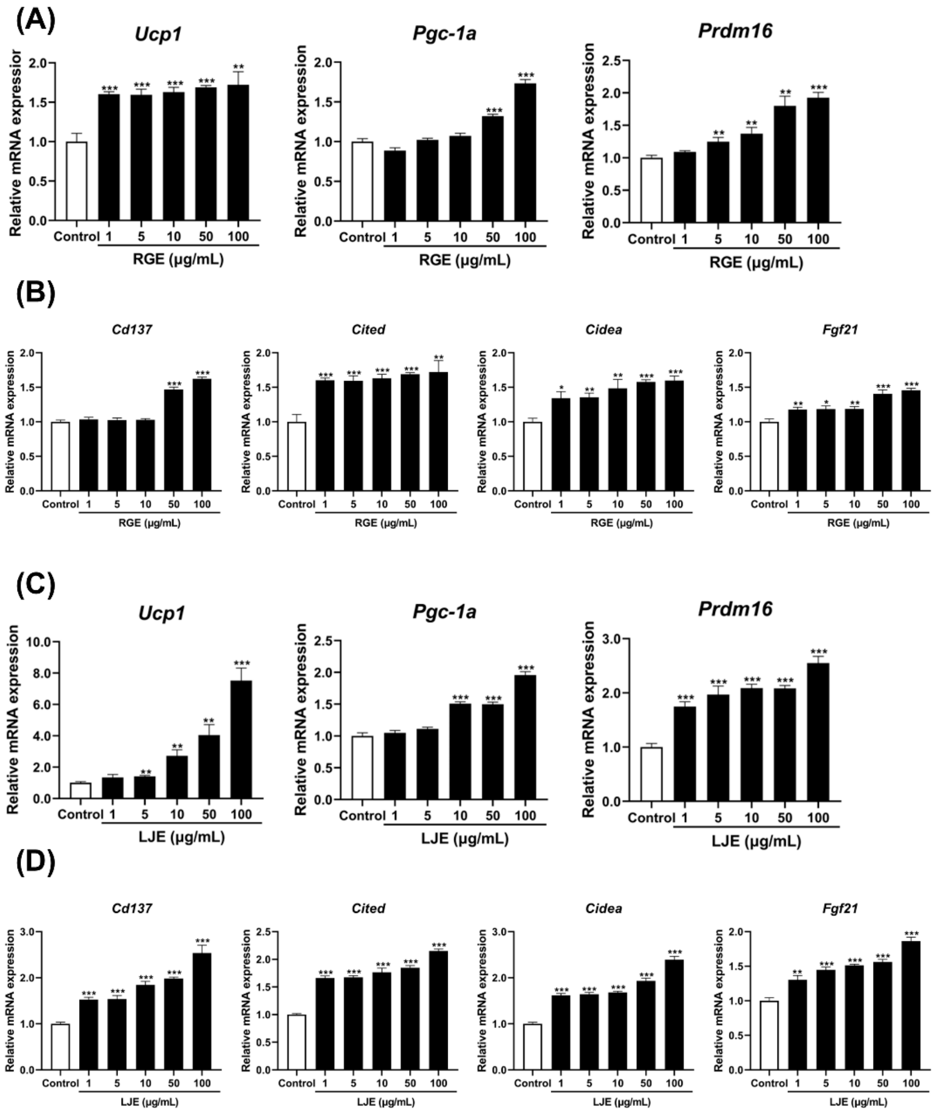

Figure S2. Effects of RGE (A, B) and LJE (C, D) on the expression of thermogenic and beige fat-specific markers in 3T3-L1 adipocytes. Target gene mRNA levels were normalized to *Gapdh* using the 2–ΔΔCt method (n = 6). Gapdh was used as the housekeeping gene. Results are presented as mean ± standard error of the mean (SEM). * $p < 0.05$, ** $p < 0.01$, and *** $p < 0.001$ vs. control.

Figure S3, Supplementary Data

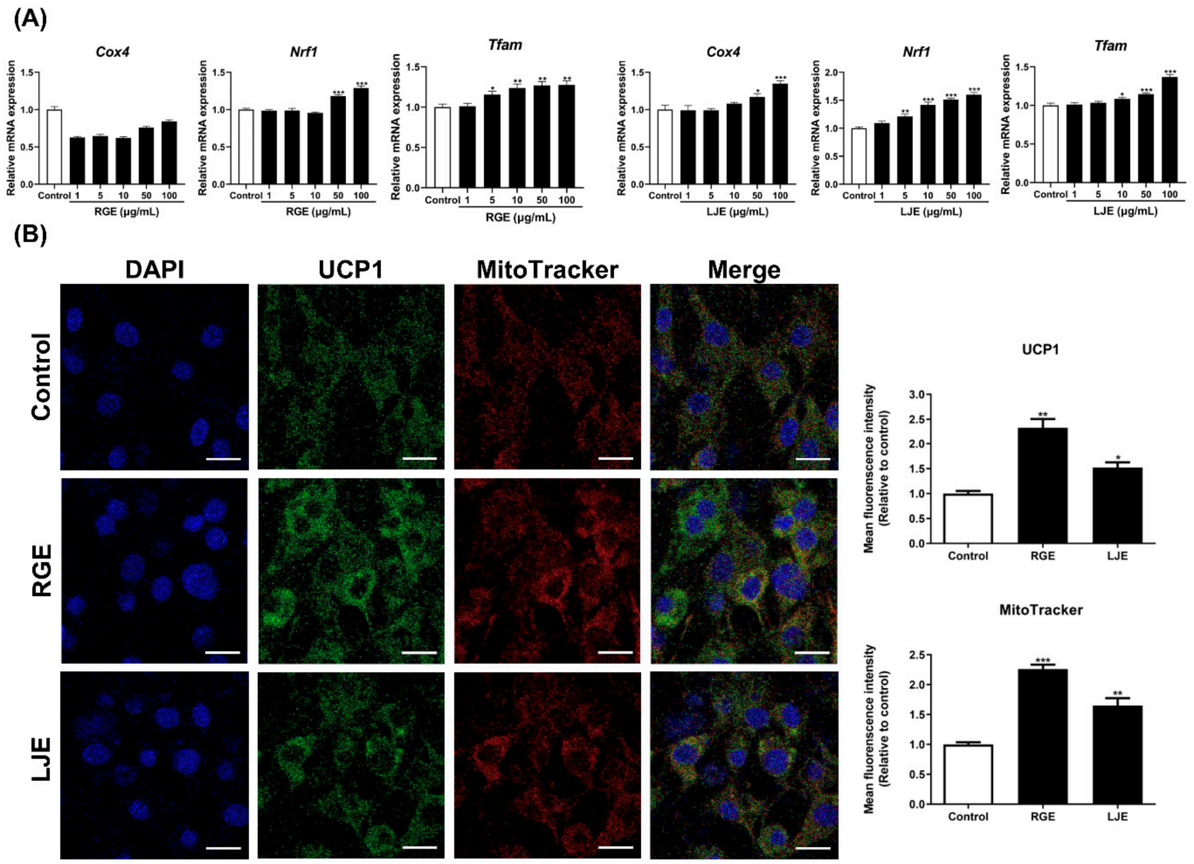

Figure S3. Effects of RGE and LJE on mitochondrial biogenesis in 3T3-L1 adipocytes. (A) mRNA expression of mitochondrial biogenesis markers was evaluated using qRT-PCR. *Gapdh* was used as the housekeeping gene, and target gene expression was normalized using the 2−ΔΔCt method. Results are presented as mean ± standard error of the mean (SEM) ($n = 6$). * $p < 0.05$, ** $p < 0.01$, and *** $p < 0.001$ vs. control. (B) Effects of RGE and LJE on intracellular mitochondrial biogenesis, with UCP1 activation evaluated using immunofluorescence staining ($n = 3$). UCP1 protein localization was visualized using FITC-conjugated antibody (UCP1-FITC), DAPI (nuclei), and MitoTracker Red (mitochondria). Images were obtained at 60× magnification (scale bar = 10 μm).

Figure S4, Supplementary Data

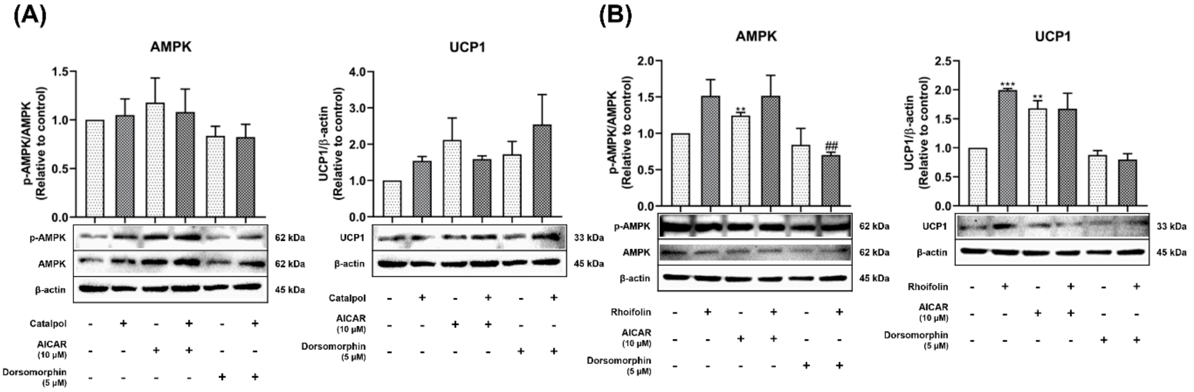

Figure S4. Effects of catalpol and rhoifolin on the expression of fat browning through AMPK signaling pathway in 3T3-L1 adipocytes. β-actin was used as a loading control for protein analysis. Cells were co-treated with catalpol (A) or rhoifolin (B) along with an AMPK activator (AICAR, 10 μM) or AMPK inhibitor (dorsomorphin, 5 μM). Results are presented as mean ± standard error of the mean (SEM) ($n = 3$). ** $p < 0.01$, and *** $p < 0.001$ compared with the control (increase); ## $p < 0.01$ compared with the control (decrease).

Figure S5, Supplementary Data

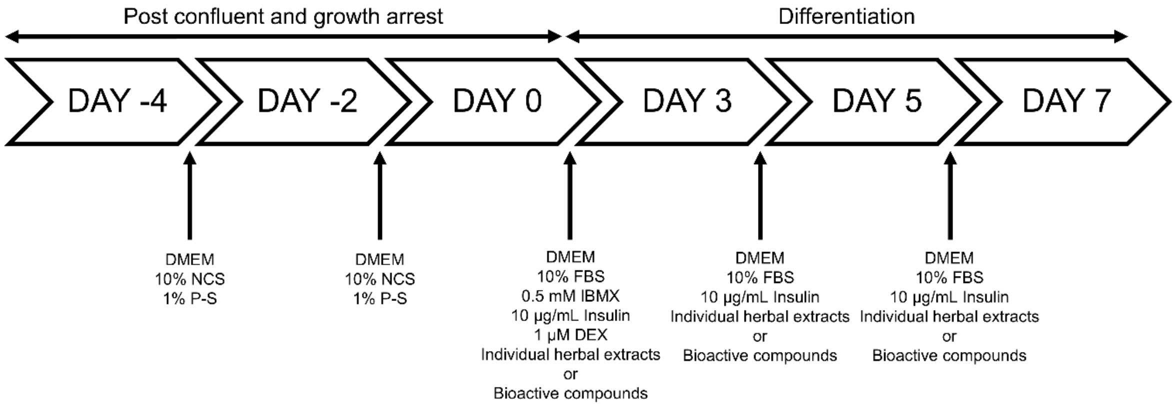

Figure S5. 3T3-L1 cell differentiation process.
